# Systematic Comparison of Three Methods for Fragmentation of Long-Range PCR Products for Next Generation Sequencing

**DOI:** 10.1371/journal.pone.0028240

**Published:** 2011-11-30

**Authors:** Ellen Knierim, Barbara Lucke, Jana Marie Schwarz, Markus Schuelke, Dominik Seelow

**Affiliations:** 1 NeuroCure Clinical Research Center (NCRC), Charité – Universitätsmedizin Berlin, Berlin, Germany; 2 Department of Neuropediatrics, Charité – Universitätsmedizin Berlin, Berlin, Germany; Natural History Museum of Denmark, Denmark

## Abstract

Next Generation Sequencing (NGS) technologies are gaining importance in the routine clinical diagnostic setting. It is thus desirable to simplify the workflow for high-throughput diagnostics. Fragmentation of DNA is a crucial step for preparation of template libraries and various methods are currently known. Here we evaluated the performance of nebulization, sonication and random enzymatic digestion of long-range PCR products on the results of NGS. All three methods produced high-quality sequencing libraries for the 454 platform. However, if long-range PCR products of different length were pooled equimolarly, sequence coverage drastically dropped for fragments below 3,000 bp. All three methods performed equally well with regard to overall sequence quality (PHRED) and read length. Enzymatic fragmentation showed highest consistency between three library preparations but performed slightly worse than sonication and nebulization with regard to insertions/deletions in the raw sequence reads. After filtering for homopolymer errors, enzymatic fragmentation performed best if compared to the results of classic Sanger sequencing. As the overall performance of all three methods was equal with only minor differences, a fragmentation method can be chosen solely according to lab facilities, feasibility and experimental design.

## Introduction

In the last few years Next Generation Sequencing (NGS) technologies have fundamentally changed genomic research and have opened up many new research areas and novel diagnostic applications. Targeted sequencing of all human exons has already led to the discovery of many genetic defects, providing new insights into the pathophysiology of inherited diseases [1]–[3]. These larger projects were all performed in dedicated, highly specialized centers for genomic research. However, NGS technology recently also entered the realm of routine diagnostics, demanding a further streamlining and simplification of the sample processing pipeline. Common to most sample preparation protocols, the starting material consists of high molecular weight double-stranded DNA that has to be fragmented.

One commonly used method is DNA shearing *via* nebulization. Compressed nitrogen or air forces input DNA repeatedly through a small hole producing random mechanically sheared fragments leading to a heterogeneous mix of double-stranded DNA molecules containing 3′- or 5′ overhangs as well as blunt ends. Sonication is another method to fragment DNA. Samples are subjected to ultrasonic waves, whose vibrations produce gaseous cavitations in the liquid that shear or break high molecular weight DNA molecules through resonance vibration. Finally, enzymatic DNA digestion might be an effective alternative to the random shearing methods. Recently, a commercial enzymatic fragmentation kit (NEBNext™, New England Biolabs, Ipswich, MA) has become available, which generates random DNA fragments between 100 and 800 bp length, depending on the incubation time. NEBNext™ dsDNA Fragmentase is a mix of two enzymes, one randomly generating nicks in the dsDNA and the other one cutting the strand opposite to the nicks. The ensuing dsDNA fragments contain short overhangs, as well as 5′-phosphates and 3′-OH-groups. Finally, single stranded overhangs and DNA fragments that have been nicked but not cut on the opposite strand have to be repaired by *Escherichia coli* DNA ligase.

The transfer of NGS technologies into the routine diagnostic setting has to address several key issues such as the simplification and multiplexing of sample preparation, as well as the streamlining of bioinformatic analysis. In a diagnostic setting, for example, the comprehensive search for Mendelian mutations in disease genes should ideally comprise the entire gene including promoter regions, 5′- and 3′-UTR, exons as well as introns. Using classic Sanger sequencing, such an effort is often too laborious and expensive for routine clinical diagnostics. The combination of long-range PCR with NGS now offers the possibility to perform such analyses in a time-efficient and economical way. We applied this method for the analysis of the *LPPR4* gene (syn. *PRG1*, MIM *607813) in patients with epilepsy [4].

As there was only little information in the literature on the advantages and disadvantages of different fragmentation methods, we set out to determine the optimum method for such high-throughput sequence analysis and evaluated the three above mentioned fragmentation methods in a controlled and systematic manner. For that we prepared three separate template libraries for each fragmentation method with three technical replicates, “barcoded” them and sequenced them jointly during the same run on a Roche GS Junior Sequencer (Roche, Branford, CT) using the 454 pyrosequencing technology. Finally, we evaluated sequence coverage, read qualities, as well as error rates of all discovered sequence variants and compared those to the results of Sanger sequencing serving as the “gold standard”.

## Results and Discussion

### Sequencing and alignment

**Figure 1:**
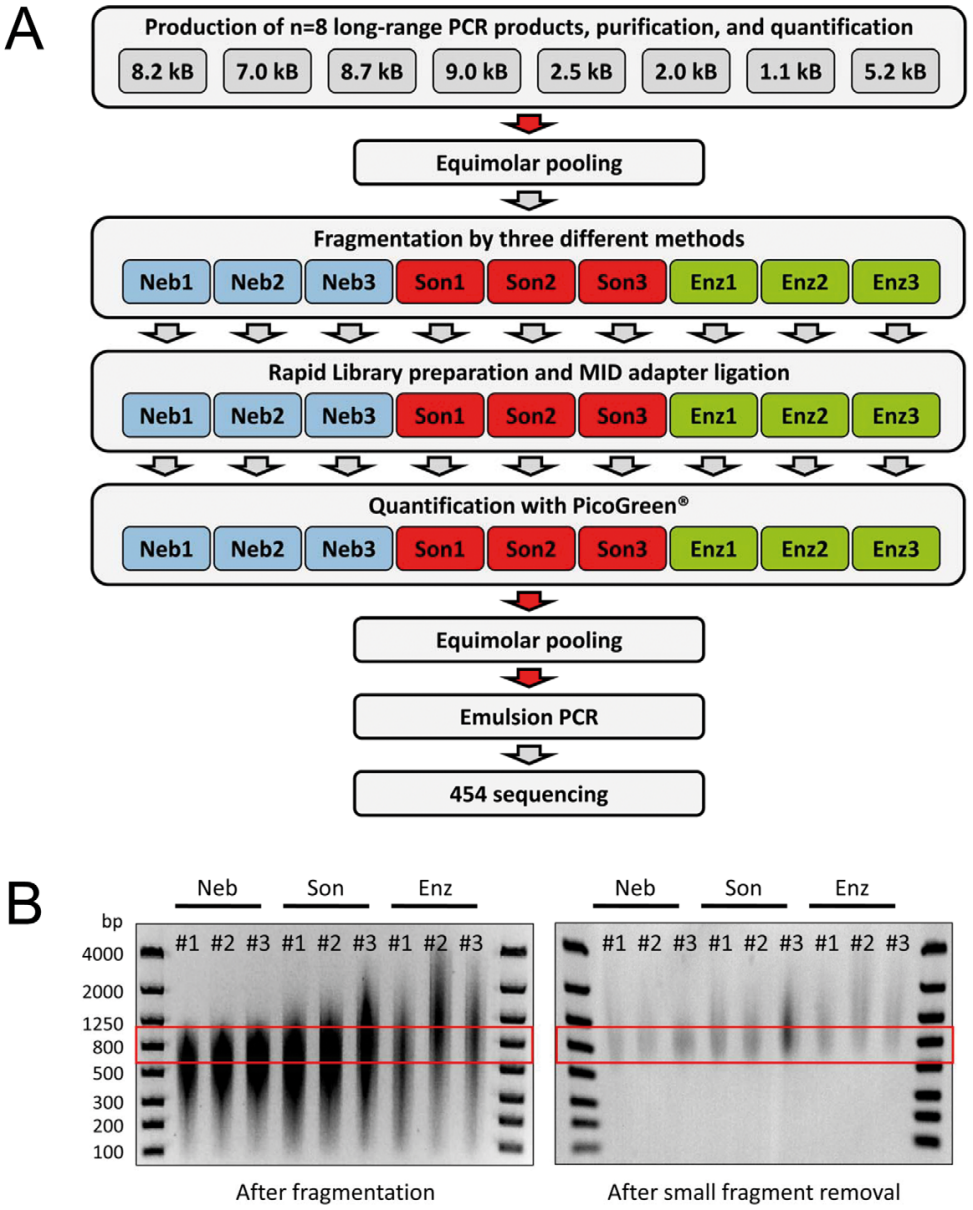
Workflow for fragmentation and NGS sequencing of long-range PCR fragments. (**A**) Graphical illustration of the entire workflow. The red arrows depict a measuring and DNA-quantification step. (**B**) Analysis of fragment lengths by PAGE before (left panel) and after (right panel) removal of small fragments <500 bp with AMPure™ columns. The red boxes depict the desired size range between 600 and 1,000 bp. Neb, nebulization; Son, sonication; Enz, enzymatic fragmentation.

For each of the three methods we prepared three sequencing libraries according to Roche standard protocols, introduced MIDs and sequenced them in one run on the Roche GS Junior Sequencer. In total, we generated 132,207 reads, which passed the internal GS Junior quality filter, comprising approximately 54,000,000 bp of DNA sequence. In 128,696 (97,5%) of those reads, the introduced MIDs could be rediscovered, comprising 49,052 reads of the nebulization (Neb), 42,403 reads of the sonication (Son) and 37,515 reads of the enzymatic (Enz) fragmentation group. 3,238 (2.5%) of all reads could not be allocated to an MID. The median fragment length was 455 bp (Neb), 451 (Son), and 441 bp (Enz); the mean[±SD] fragment length was 410[±120] (Neb), 407[±121] (Son), 397[±126] (Enz) and the mode of the fragment length was 491 (neb), 496 (Son), and 490 (Enz). We did not find any significant differences between the methods (Figure 1C).

**Figure 2:**
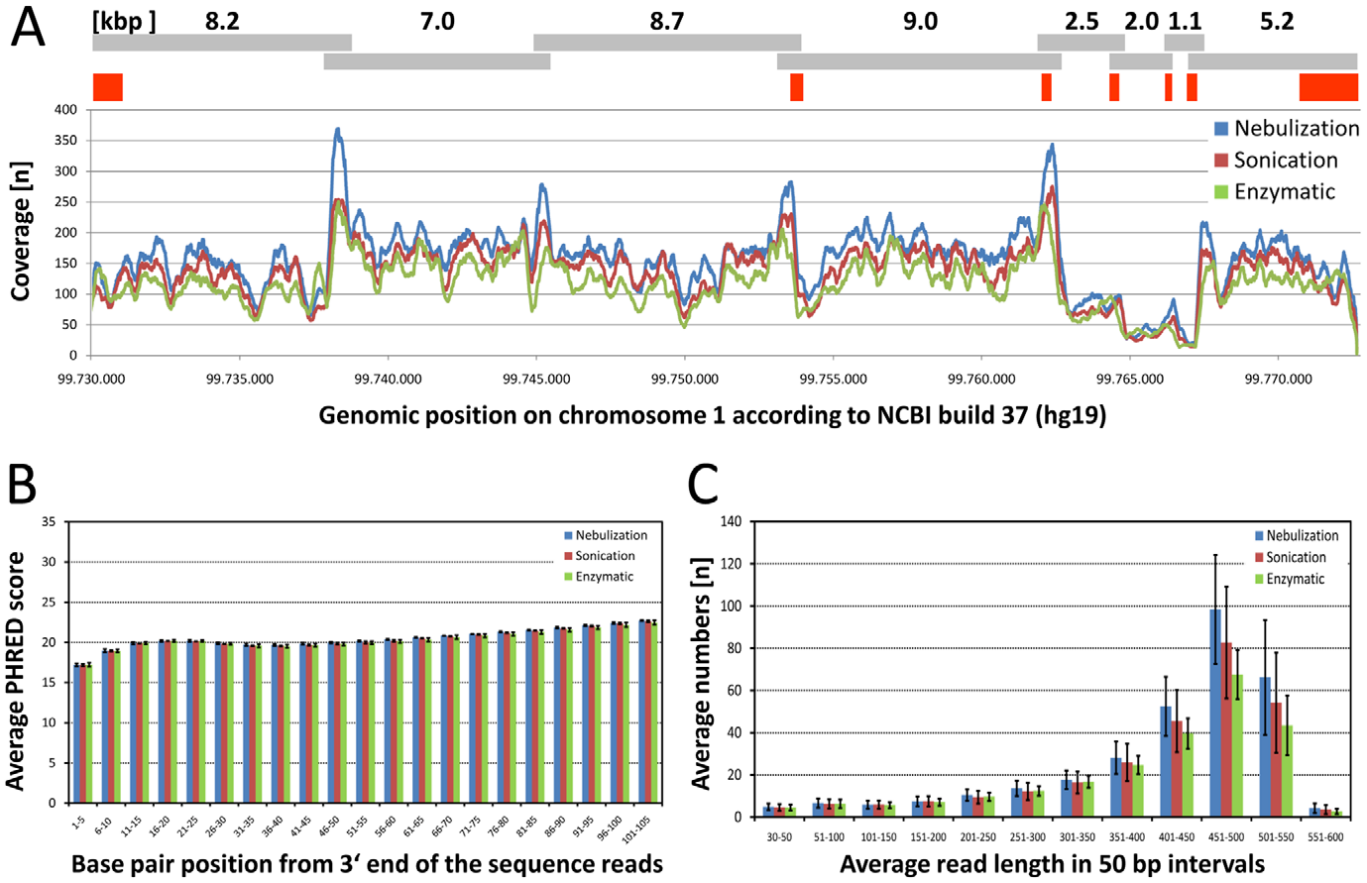
Coverage, sequence quality and read lengths of the 454 sequence run. (**A**) Sequence coverage over the entire genomic region of the *LPPR4* gene. The colors separate the results with respect to the fragmentation method. The gray bars above the graph depict the location and length PCR fragments [in kbp] and the red squares highlight the seven coding exons of the ENST00000370185 transcript. It becomes clear that the sequence coverage drops considerably for all three fragmentation methods if the PCR fragment size is below 3,000 bp. (**B**) Comparison of the sequence qualities scores (PHRED) at the 3′-ends of the sequences that have been generated using the three fragmentation methods. The bars depict the mean and standard deviation for three replicates of each fragmentation method, averaged over stretches of 5 base pairs. No significant difference was found between the three fragmentation methods. (**C**) Number of the sequence reads for different read lengths (averaged over stretches of 50 base pairs). The error bars depict the standard deviation of three replicates for each fragmentation method. This shows the variation between technical replicates to be larger than between the averages of the three fragmentation methods. No significant difference was found between the three methods.

Complete coverage of the target region was achieved with all three fragmentation methods (Figure 2A). Among the experimental groups we found nearly the same percentage of reads that could not be aligned to the reference sequence. The number of aligned sequence reads for each individual experiment, however, varied more inside the three technical replicates than between the different experimental groups (Figure 2C). Such variance is likely to be caused by minor inconsistencies during fluoroscopic measurement and pipetting of the minute DNA quantities before final pooling for emulsion PCR, finally leading to the unequal representation of nine individual libraries in the final sequence readout.

Similar for all three sample preparation methods, we found an uneven coverage across our region of interest. We assume this to be a result of our experimental design. The length of our long-range PCR fragments lay between 1 to 9 kbp (Figure 2A). The poorly covered 5.2 kbp region between chr1:99,762,380-99,767,573 was amplified by long-range-PCR in three smaller amplicons of 2.5 kbp, 2.0 kbp and 1.2 kbp. In contrast to the larger fragments, which comprise approximately 8–9 kbp, fragmentation of the smaller fragments likely resulted in a disproportionate larger amount of fragments below 500 bp, which were subsequently removed by the small fragment removal procedure. Consequently, the fragment coverage of this region dropped proportionally to the length of the PCR products and led to an under-representation of this area in the sequence realignment. Thus, if using long-range PCR products with the above mentioned protocol, one should strive (1) to keep the long-range PCR product sizes >3,000 bp and (2) to design PCR products that are in the same size range.

### Comparison of reads qualities

As different fragmentation protocols might carry over chemicals/enzymes from preceding steps into library preparation and sequencing reactions that in turn might affect read qualities, we analyzed the quality scores specifically at the 3′-ends of the sequence fragments (Figure 2B). The Roche GS Junior base calling software provides PHRED quality scores for each base. PHRED scores provide a sequencing error estimate and are hence a good tool to assess the quality of sequences and to compare the reliability of different sequencing runs on the same instrument [5]. We did not detect any significant differences in the quality scores obtained with the three different fragmentation methods (Figure 2B). For each method, the quality score continuously fell from the beginning to the end of the sequence. The choice of a particular fragmentation method did not have any influence on sequence quality as reflected by the PHRED score.

### Comparison of error rates

Physical as well as enzymatic fragmentation methods might introduce DNA damage other than double strand breaks, e.g. closely spaced DNA nicks or loss of nucleotides on one strand with subsequent gap repair resulting in short deletions. We had seen such errors especially with the enzymatic fragmentation method if insufficient amounts of ligase were added.

**Figure 3:**
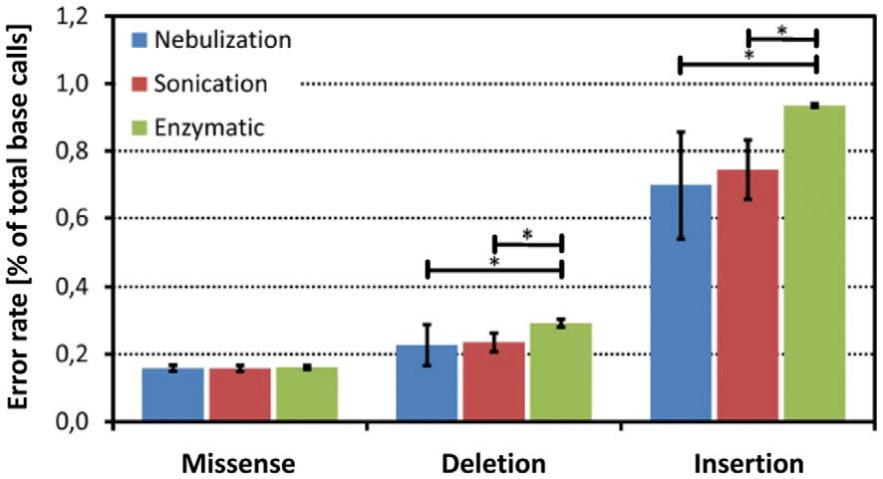
Comparison of the percentage of missense, deletion and insertion errors in individual sequence reads. The error frequency was calculated according to Method#1 (see Materials and Methods section) with respect to the fragmentation method. The error bars depict the standard deviation. In order to classify a position on a sequence read as erroneous, the coverage of the respective position had to be >20 fold and the percentage of the alternative (erroneous) allele to be <20%. *, p<0.05.

For missense errors we did not find any significant difference of error rates between the three fragmentation methods (Figure 3). With regard to the rate of insertion and deletion errors, the nebulization and the sonication groups did not show any difference. Both methods performed better (p<0.05) than enzymatic fragmentation (Figure 3). It is, however, noteworthy that the errors radically dropped when filters (e.g. for homopolymer errors), were applied during the variant calling procedure. Homopolymer sequencing errors – which may lead to false InDel calls – are a well-known problem of the Roche 454 technology and result from the nonlinear increase of luminescence during pyrosequencing of longer homopolymers [6]. Enzymatic digestion seems to slightly worsen this problem. A possible explanation being that during the random nicking and nick-repair procedures DNA material is lost or subjected to faulty end-repair by the *Escherichia coli* ligase.

In order to evaluate the quality of the final base calls, we analyzed four heterozygous SNPs that had been discovered by Sanger sequencing and a further three positions where NGS base calls deviated from homozygous Sanger base calls in at least one experiment or technical replicate (Table 1). In this analysis we found base call errors only in the nebulization and sonication subgroups. These comprised mainly insertions/deletions. For the heterozygous positions, only one genotype was wrongly predicted in the sonication group, most probably due to low coverage (only n = 4 fragments covered this position). In the seven positions assessed, we did not find any incorrect genotype prediction for the enzymatically digested samples. Taken together, all nine subgroups achieved 100% correct genotype predictions for all positions.

**Table 1:** NGS results in comparison with classic Sanger sequencing.

Genomic position on Chr 1 (hg19)	RefSeq allele	Sequencing(Sanger)		ALL	Nebulization (Neb)			Sonication (Son)			Enzymatic (Enz)		
					Exp#1	Exp#2	Exp#3	Exp#1	Exp#2	Exp#3	Exp#1	Exp#2	Exp#3
99.748.311	T	het	T|G	T|GΣ 753T = 51%	T|GΣ 44T = 59%	T|GΣ 143T = 52%	T|GΣ 71T = 52%	T|GΣ 150T = 51%	T|GΣ 36T = 64%	T|GΣ 96T = 47%	T|GΣ 98T = 55%	T|GΣ 53T = 43%	T|GΣ 62T = 40%
99.748.324	C	het	C|T	C|TΣ 729C = 63%	C|TΣ 49C = 67%	C|TΣ 140C = 65%	C|TΣ 71C = 69%	C|TΣ 144C = 57%	C|TΣ 35C = 69%	C|TΣ 85C = 61%	C|TΣ 87C = 71%	C|TΣ 55C = 65%	C|TΣ 63C = 52%
99.748.522	A	het	A|G	A|GΣ 779A = 51%	A|GΣ 62A = 60%	A|GΣ 151A = 52%	A|GΣ 78A = 54%	A|GΣ 157A = 46%	A|GΣ 43A = 49%	A|GΣ 102A = 53%	A|GΣ 75A = 53%	A|GΣ 49A = 49%	A|GΣ 62A = 45%
99.762.338–99.762.339	AA	hom	AA|AA**	AA|AAΣ 1221AA = 99%	**AA|A[]**Σ **157** **AA = 74%**	AA|AAΣ 228AA = 100%	AA|AAΣ 146AA = 98%	AA|AAΣ 205AA = 99%	**AA|A[]** **Σ 54** **AA = 72%**	AA|AAΣ 160AA = 100%	AA|AAΣ 123AA = 99%	AA|AAΣ 99AA = 99%	AA|AAΣ 121AA = 99%
99.764.728	T	hom	T|T	T|TΣ 482T = 100%	T|TΣ 22T = 100%	T|TΣ 109T = 100%	T|TΣ 62T = 100%	T|TΣ 84T = 99%	**T|TA** **Σ 16** **T = 75%**	T|TΣ 67T = 100%	T|TΣ 42T = 100%	T|TΣ 42T = 100%	T|TΣ 37T = 97%
99.767.383	C	het	C|G	C|GΣ 138C = 49%	C|GΣ 13C = 31%	C|GΣ 29C = 59%	C|GΣ 21C = 29%	C|GΣ 16C = 56%	**C|C** * **Σ 4** **C = 50%**	C|GΣ 22C = 45%	C|GΣ 11C = 64%	C|GΣ 12C = 50%	C|GΣ 10C = 60%
99.772.437–99.772.438	TT	hom	TT|TT**	TT |TTΣ 675TT = 100%	TT|TTΣ 37TT = 100%	TT|TTΣ 109TT = 100%	TT|TTΣ 132TT = 100%	TT|TTΣ 109TT = 100%	TT|TTΣ 22TT = 100%	**TT|T[]** **Σ 98** **TT = 77%**	TT|TTΣ 82TT = 99%	TT|TTΣ 54TT = 99%	TT|TTΣ 78TT = 100%

The seven out of 4,096 analyzed positions comprise four heterozygous SNPs as well as three homozygous positions that had been misinterpreted by at least one experiment. 1^st^ line: NGS sequence call after alignment and homopolymer filtering; 2^nd^ line: Σ, number of sequence fragments covering the respective position; 3^rd^ line: percentage of the calls for the major allele. Erroneous positions are highlighted in bold face.

*SAMtools prints the allele counts before it applies the homopolymer filter but calls the genotype afterwards. Allele frequencies and predicted genotype may hence differ.

**Please note that SAMtools only reports InDels supported by a sufficient number of reads. For the subsets in which this was not the case, we calculated the percentage of the RefSeq allele with all counts for both positions and display the mean coverage over both positions as Σ.

In conclusion all three methods are capable to generate high quality sequencing libraries for 454 Next Generation Sequencing. However, if the long-range PCR products are equimolarly pooled, subsequent coverage drastically drops in fragments smaller the average length (in our case <3,000 bp). We therefore suggest keeping a uniform sequence length if long-range PCR fragments are used. All three methods performed equally well with regard to overall sequence quality (PHRED) and read length. Enzymatic fragmentation showed highest consistency between three library preparations but performed slightly worse than sonication and nebulization with regards to insertions/deletions in the raw sequence reads. However, after filtering for homopolymer errors enzymatic fragmentation performed best if compared to the Sanger sequencing results. As the overall performance of all three methods was equal with only minor differences, a fragmentation method can be chosen solely according to lab facilities, feasibility and experimental design.

## Materials and Methods

### Long-range-PCR and DNA fragmentation

The study was approved by the Institutional Review Board of the Charité (Reference no. EA1/215/08) and written informed consent according to the Declaration of Helsinki was obtained from the study subject. The entire workflow is depicted in Figure 1A. Genomic DNA was isolated from peripheral human blood cells by salt extraction. Oligonucleotide primers for long-range PCR were designed using Primer3Plus [7] (sequences are available on request) and ordered from MWG (Ebersberg, Germany). Eight overlapping fragments ranging between 1,000 and 9,000 bp, covering the entire *LPPR4* gene, were amplified from a genomic DNA sample. PCR was performed with a long-range protocol using the Expand Long-Range kit (Roche) that facilitates consistent amplification of extra-long templates of up to 20 kbp. The purified amplicons were quantified using a NanoDrop™ photometer (Peqlab, Erlangen, Germany) and subsequently pooled in equimolar amounts. We thus created nine test samples, three for each fragmentation method. Each of the **Neb1-3** samples was individually fragmented by nebulization using compressed nitrogen gas and the GS FLX Titanium Rapid Library Preparation Kit (Roche). Nebulization was performed for the duration of one minute with nitrogen (pressure 2.1 bar) with a total input of 750 ng DNA for each sample. The samples **Son1-3** were each individually sonicated with 750 ng DNA in a reaction volume of 100 µl using the Bioruptor™ (Diagenode, Liège, Belgium) with the following settings: five sonication cycles (30 sec ON, 30 sec OFF) to obtain the desired DNA fragment size between 600–1,000 bp. The temperature was kept at 4°C. The samples **Enz1-3** were subjected individually to enzymatic fragmentation using the NEBNext™ dsDNA Fragmentase kit (NEB) according to the manufacturer's instructions. Briefly, a total of 750 ng DNA from each sample was complemented with the necessary components to a total volume of 20 µl. The optimum incubation time of 35 min at 37°C had been empirically determined in a time series to aim for a fragment size between 600–1,000 bp. The resulting size distribution for each fragmentation method before and after small fragment removal was controlled by gel electrophoresis (Figure 1B).

### Template library preparation, emulsion PCR and pyrosequencing

For each fragmented sample a GS Junior DNA library was prepared according to the protocol of the manufacturer (Rapid Library Preparation Method, Roche). Fragmented DNA was subsequently end-repaired and phosphorylated using T4 DNA polymerase and T4 polynucleotide kinase. The nine different samples were then labeled *via* ligation of Multiplex Identifiers (MID) oligonucleotide adaptors. These unique MID-sequences are located at the 5′-end of each sequence read and provide a “barcode” to later re-identify and assign the sequences to the respective test samples. Hence, we were able to pool all nine samples into a single sequencing run, thus preventing systematic errors that might have been introduced in separate sequencing runs. Small DNA fragments were subsequently removed using the AMPure™ PCR purification system (Agencourt Bioscience, Bernried, Germany) and library quality was assessed using a FlashGel™ System (Lonza, Basel, Switzerland) (Figure 1B). Finally, the nine different libraries were quantified and equal amounts were pooled into a single sample. Adapter-modified fragments were diluted, annealed to capture beads, and clonally amplified by emulsion PCR (emPCR, Amplification Method Manual - Lib-L; GS Junior Titanium Series, Roche). After emPCR, beads with the cloned amplicons were enriched, loaded onto the 454 picotiter plate, and sequenced on the Roche GS Junior Sequencer according to the manufacturer's protocol (Sequencing Method Manual, GS Junior Titanium Series, Roche).

### Data analysis

Image analysis and base calling of the raw sequencing data were performed using the default “shot-gun” Roche GS Junior data analysis pipeline. The sequence reads were aligned to the reference sequence of human chromosome 1 (hg 19/build 37) using BWA-SW, an algorithm designed for long reads with more errors [8]. The alignment was carried out with default parameters. Data processing for variant calling and error statistics was done with SAMtools/bcftools [9], the *mpileup* function was used with the homopolymer filter option (−h) set to 5 and the region (−r) restricted to the coordinates of *LPPR4* (chr1:99,725,000-99,778,000).

### Sanger sequencing

Genomic DNA was isolated from peripheral blood cells by salt extraction. PCR oligonucleotide primers covering all 7 coding exons of the ENST00000370185 transcript of the *LPPR4* gene, including 50 bp flanking intronic regions, were designed using GeneDistiller [10] and ordered from MWG (oligonucleotide primer sequences are available on request). PCR fragments were sequenced into both directions using the BigDye™ Terminator 3.1 protocol (Applied Biosystems, Darmstadt, Germany) on a ABI3730 capillary sequencer according to standard protocols. Sequences were analyzed using MutationSurveyor v3.10 (Softgenetics, State College, PA, USA).

### Sequencing error assessment

We compared the error rates of the three fragmentation methods thereby discriminating between missense and insertion/deletion (InDel) errors. We employed two different methods to evaluate those errors, which were both applied *after* variant calling. **Method#1** is a statistical algorithm to compare the true error rate in the “raw” sequences. We explicitly did not apply any filters to the variant detection and switched off those active by default, e.g. to exclude low-quality or rare bases. As we did not know the “true” DNA sequence of the sample over the entire range, we focused on positions with a coverage depth of 20 or higher and an unambiguous genotype (frequency of the alternative allele below 20%). For all positions we considered those bases equal to the reference sequence or the major allele as “correct” reads and all other bases as “false” reads. Errors for single base exchanges and InDel variants were counted separately. To finally compare the different fragmentation methods, the numbers of respective correct and false bases were added over the entire reference sequence and given as a percentage of the total base calls.

For **Method#2** of sequencing error assessment we used Sanger sequencing data, which were available for 4,096 bp positions covering all exons and flanking intronic regions of our gene of interest. For these positions we compared the NGS data with the observed bases in the Sanger sequences. Here we used the NGS variant prediction by SAMtools of base calls *after* filtering for rare variants, most of them homopolymer sequencing errors. If the NGS predicted genotype differed from that observed in the Sanger sequencing, we considered this an erroneous read (Table 1).
